# Fish oil supplements, longevity and aging

**DOI:** 10.18632/aging.101021

**Published:** 2016-08-25

**Authors:** João Pedro de Magalhães, Michael Müller, G. Ed. Rainger, Wilma Steegenga

**Affiliations:** ^1^ Integrative Genomics of Ageing Group, Institute of Ageing and Chronic Disease, University of Liverpool, Liverpool, United Kingdom; ^2^ Norwich Research Park Food and Health Alliance, Norwich, United Kingdom; ^3^ Centre for Cardiovascular Sciences, School of Clinical and Experimental Medicine, The Medical School, The University of Birmingham, Birmingham, United Kingdom; ^4^ Division of Human Nutrition, Wageningen University and Research Centre, Wageningen, The Netherlands

**Keywords:** fish oil, health, longevity, mice, omega-3 fatty acids

## Abstract

Fish oil supplementation is of great medical and public interest with epidemiological evidence of health benefits in humans, in particular by conferring protection against heart diseases. Its anti-inflammatory properties have also been reported. Initial results from short-lived mouse strains showed that fish oil can increase lifespan, affecting pathways like inflammation and oxidation thought to be involved in the regulation of aging. Could fish oil and its omega-3 fatty acids act as geroprotectors? Probably not. A new study by Strong et al. challenges the role for fish oil supplementation in aging. Using a large cohort of genetically heterogeneous mice in three sites, part of the Interventions Testing Program of the NIA, Strong et al. show that fish oil supplementation at either low or high dosages has no effect on the lifespan of male or female mice. Although it is still possible that fish oil supplementation has health benefits for specific age-related diseases, it does not appear to slow aging or have longevity benefits.

An increasing number of people are turning to supplements of fish oil and of its omega-3 poly-unsaturated fatty acids eicosapenatenoic acid (EPA) and docosahexaenoic acid (DHA). These fatty acids can only be synthesized in mammals from the dietary precursor and essential fatty acid, α-linolenic acid [1]. However, the synthesis pathway requires a number of elongation and desaturation steps, making direct uptake from the diet a more effective route of assimilation. The EPA and DHA in the human diet are derived indirectly from marine algae (higher plants lack the enzymes for the biosynthesis of these lipids) and their bioavailability is dramatically increased as they pass up the food chain and are concentrated in the flesh of marine fish. Sales of omega-3 supplements have been growing steadily and are valued at over 1 billion dollars in the US alone [2], and omega-3 products in general are worth many billions in sales every year.

The growing public and clinical interest in fish oil and its omega-3 fatty acids is not surprising considering the large number of studies reporting health benefits of fish, fish oil and omega-3 fatty acids consumption. Triggering interest in fish oil were studies conducted in Greenland Eskimos showing that large amounts of fish consumption protected against heart disease, in spite of a large intake of fat and cholesterol [3–6]. Since then several epidemiologic studies have associated reduced risk of cardiovascular disease with fish or fish oil consumption [7–10]. Furthermore, one study in coronary heart patients found that blood levels of omega-3 fatty acids were inversely associated with telomere shortening [11]. Fish oil has been shown to impact on several risk factors associated with coronary heart disease [3]. Briefly, fish oil lowers triglyceride levels [9]. This has been observed in human studies in a dose-response manner, accompanied by increases in LDL cholesterol (and to a lower degree HDL cholesterol) levels [12]. Both EPA and DHA, in fact, have triglyceride-lowering properties [13]. In patients with mild hypertension, but not in healthy subjects, consumption of omega-3 fatty acids has been shown to decrease systolic and diastolic blood pressures [14]. One hypothesis is that, by modulating inflammation within the artery wall, omega-3 fatty acids also alter the structural composition of advanced atherosclerotic plaques in a manner that could reduce the incidence of plaque rupture or ulceration, a process that precedes tissue infarction (e.g. heart attack or stroke) [15].

Although the benefits of fish oil have been more widely studied in the context of heart disease, studies suggest that consumption of fish oil or of its omega-3 fatty acids may have beneficial effects on stroke, depression, diabetes mellitus, cancer and Alzheimer's disease [3,5,16,17]. Because dietary omega-3 polyunsaturated fatty acids have anti-inflammatory properties, their beneficial effects have been reported for pathologies and conditions associated with inflammation. For example, oral supplements containing omega-3 fatty acids reduced symptoms and clinical scores associated with inflammatory activity in psoriasis [18]. Similarly, fish oil consumption has been shown to reduce the symptoms of disease as well as the use of non-steroidal anti-inflammatory drugs in arthritis patients with severe inflammatory joint disease [19]. One study showed that fish oil supplementation induces anti-inflammatory gene expression profiles in human blood mononuclear cells [20]. In vitro studies have also suggested that omega-3 fatty acids have direct effects on inflammatory responses [21–23].

Numerous studies in mice have supported the beneficial roles of fish oil consumption. Succinctly, in mice, fish oil may have beneficial effects on arthritis [24], cancer [25], cardiac arrhythmias [26] and on bone mass during aging [27]. In murine models of atherosclerosis, the burden of arterial plaque was reduced upon omega-3 fatty acid supplementation [28]. One study reported beneficial effects of dietary omega-3 polyunsaturated fatty acids on toxin-induced neuronal degeneration in an animal model of Parkinson's disease [29].

Fish oil has been shown to increase lifespan by over 40% in autoimmune-prone (NZB x NZW)F(1) female mice [30–33]. Lifespan extension in this NZB/W strain was accompanied by decreased body weight and lowered inflammation levels such as lower NFkB. Interestingly, enhanced antioxidant enzyme (superoxide dismutase, catalase and glutathione peroxidase) activities were also observed and may partly explain the lower NFkB levels [30,31]. Moreover, a demographic analysis of these fish oil-fed NZB/W revealed that fish oil could shift the Gompertz slope, suggesting that fish oil may delay the rate of aging in NZB/W mice [34]. Another study in mice found evidence of increased antioxidant gene expressions in response to fish oil, though it is possible that this is a defense mechanism since fish oil is easily peroxidized [35]. As such, fish oil affects pathways thought to be involved in the regulation of aging, making it a candidate geroprotector. There is enormous interest in discovering and developing geroprotectors [36], and given that a significant number of people already self administer omega-3 fatty acids for health reasons, evaluating if fish oil is a geroprotector is of great scientific and public interest.

For the above reasons, we proposed fish oil to the Interventions Testing Program (ITP) of the NIA, which investigates the effects of treatments or compounds on the lifespan and aging of mice. Our proposal was approved and two dosages were tested across three sites using a large cohort (n = 287 males and 267 females) of genetically heterogeneous mice. The results reveal no significant longevity benefits of fish oil supplementation of either dose on either sex [37]. A dose-dependent increase in body weight was observed in males at 18 months of age. There was some variation across the three sites with an 18% decline in male longevity observed for the higher dose in one site while a 9% increase in lifespan was observed for males at another site for the lower dosage [37]. Nonetheless, the pooled results do not reveal any significant effects of fish oil on mouse longevity.

Other recent murine studies also raise questions about the health and longevity benefits of fish oil. One small (n =14) study previously found no lifespan effect of fish oil supplementation in female C57BL/6 mice [38]. Another study found fish oil supplementation to shorten mean lifespan in F1 mice by 4.7 to 9.8% [39]. Because NZB/W female mice are slightly obese, autoimmune-prone, short-lived animals, the life extension caused by fish oil in NZB/W mice could be due to a delay of inflammation-triggered pathologies which are more severe in NZB/W mice. Indeed, one more recent study suggests that DHA in particular suppresses IL-18-dependent signaling and glomerulonephritis in NZB/W mice [40]. However, one study found that dietary fish oil in a mouse model of inflammatory colitis induces severe colitis and adenocarcinoma formation [41]. In another short-lived mouse model, long-term dietary fish oil decreased lifespan [42], again suggesting that fish oil's longevity benefits are limited to very specific strains. Nevertheless, given the importance of inflammation in aging [43], it is surprising that fish oil had no longevity benefits as part of the ITP.

A number of recent clinical trials also failed to substantiate the benefits of fish oil supplements. Of 18 clinical trials and 6 meta-analysis of omega-3 supplements, only 2 reported benefits [2]. Moreover, one recent study found that the indigenous people of Greenland have genetic and physiological adaptations to a diet rich in omega-3 [44]. One possibility is that the benefits of fish oil consumption depend on one's own individual genetic makeup, and some populations will have greater benefits than others. This is not unexpected given that we know that many longevity interventions depend on genome-environment interactions [45]. Although longevity benefits for fish oil supplements seem unlikely, it is still possible that fish oil supplements have a positive impact on specific age-related diseases or conditions. One of the limitations of the ITP study was that no pathological analysis was performed on the animals. Nonetheless, because mice die primarily of cancer, geroprotectors that primarily influence cancer will be more readily detected in mice [46]. One largely unexplored area concerns changes in sensitivity to supplementation with age, in particular if disease is superimposed on the aged. It may be that fish oil supplements are beneficial at some ages and for some conditions but neutral or even detrimental at others. Further studies are necessary to gain clarity on its possibility.

In conclusion, fish oil supplementation does not extend longevity in normal healthy mice. Although specific health benefits of fish oil cannot be excluded, omega-3 fatty acids and fish oil do not appear to act as geroprotectors.
